# Using *in vitro* bioassays to guide the development of safer bio-based polymers for use in food packaging

**DOI:** 10.3389/ftox.2022.936014

**Published:** 2022-09-20

**Authors:** Emma Harper, Eoin Cunningham, Lisa Connolly

**Affiliations:** ^1^ Institute for Global Food Security, School of Biological Sciences, Queen’s University Belfast, Belfast, United Kingdom; ^2^ School of Mechanical and Aerospace Engineering, Queen’s University Belfast, Belfast, United Kingdom

**Keywords:** bio-based polymers, endocrine disrupting chemicals, leachates, food packaging, steroid hormones, estrogenic, anti-androgenic

## Abstract

Petroleum-based polymers traditionally used for plastic packaging production have been shown to leach dangerous chemicals such as bisphenol-A (BPA). Bio-based polymers are potentially safer alternatives, and many can be sustainably sourced from waste streams in the food industry. This study assesses bio-based polymers undergoing food packaging development for migration of endocrine disrupting leachates at the level of estrogen, androgen and progestagen nuclear receptor transcriptional activity. Reporter gene assays were coupled with migration testing, performed using standardised test conditions for storage and temperature. Test samples include nine bio-based polymers and four inorganic waste additives mixed with a traditional petroleum-based polymer, polypropylene. Thermoplastic starch material, polybutylene succinate, polycaprolactone, polybutylene adipate terephthalate (PBAT), two polylactic acid (PLA)/PBAT blends, polyhydroxybutyrate (PHB) and eggshell/polypropylene (10:90) presented no significant reduction in metabolic activity or hormonal activity under any test condition. Polypropylene (PP) presented no hormonal activity. Metabolic activity was reduced in the estrogen responsive cell line after 10 days migration testing of eggshell/polypropylene (0.1:99.9) in MeOH at 40°C, and PP in MeOH and dH_2_0. Estrogenic agonist activity was observed after 10 days in poultry litter ash/polypropylene (10:90) in MeOH at 20°C and 40°C, poultry feather based polymer in MeOH and dH_2_O at 40°C, and eggshell/polypropylene (40:60) and PLA in dH_2_O at 40°C. Activity was within a range of 0.26–0.50 ng 17*β*-estradiol equivalents per ml, equating to an estrogenic potency of 3–∼2800 times less than the estrogenic leachate BPA. Poultry litter ash/polypropylene (10:90) in MeOH for 10 days presented estrogenic activity at 20°C and 40°C within the above range and anti-androgenic activity at 40°C. Progestagenic activity was not observed for any of the compounds under any test condition. Interestingly, lower concentrations of eggshell or PP may eliminate eggshell estrogenicity and PP toxicity. Alternatively eggshell may bind and eliminate the toxic elements of PP. Similarly, PLA estrogenic activity was removed in both PLA/PBAT blends. This study demonstrates the benefits of bioassay guidance in the development of safer and sustainable packaging alternatives to petroleum-based plastics. Manipulating the types of additives and their formulations alongside toxicological testing may further improve safety aspects.

## Introduction

Plastic packaging is used in the production, processing, transport, handling and storage of food and beverage and is of high importance as it protects food and beverage from physical damage, soiling and microbial spoilage (Muncke et al., 2017). The abundance and high resistance to degradation of plastic waste both on land and at sea has become a global concern regarding the issue of environmental pollution with plastic debris (Muncke, 2011). In addition, plastics are contributors towards increasing the effects of global warming and the depletion of non-renewable fossil resources such as oil (Nakajima et al., 2017). Petroleum-based plastics are known sources of hazardous chemicals, with chemical plasticisers such as bisphenol-A (BPA) and phthalates regularly cited for their potential hazards and epidemiological response (Zimmermann et al., 2019). BPA and phthalates can migrate out of petroleum-based plastic packaging into food, soil, water or oceans and consequently be ingested by animals or humans with the potential to cause adverse health effects (Yang et al., 2011; Moreira et al., 2015; Muncke et al., 2017; Guillard et al., 2018).

Some petroleum-based plastic leachates have also been found to cause endocrine dysfunction in humans by interfering at the point of production, release, transport, metabolism, binding or elimination of natural hormones in the body and, therefore, are recognised as endocrine disrupting chemicals (EDCs) (Yang et al., 2011; Moreira et al., 2015). EDCs are defined by the WHO/IPCS (2002) as “an exogenous substance or mixture that alters function(s) of the endocrine system and consequently causes adverse health effectsin an intact organism, or its progeny, or (sub) populations.” Human exposure to synthetic hormone mimicking chemicals such as BPA and phthalates is associated with developmental, reproductive and metabolic disrupting effects including infertility or reduced fertility, learning and memory difficulties, diabetes, obesity and cardiovascular disease (Casals-Casas, and Desvergne, 2011; Schug et al., 2011; WHO/UNEP, 2013). EDCs can affect our sex hormones leading to disruption of reproductive development, sexual differentiation, early embryonic development and puberty (Casals-Casas and Desvergne, 2011). Estrogen receptors (ERs) are one of the major targets of EDCs, thus affecting the normal estrogen signalling pathways (Shanle and Xu, 2011).

The production and use of bio-based plastics may be a potentially safer alternative to petroleum-based plastics, whilst also helping to reduce the dependency of plastics on non-renewable fossil fuels, reducing greenhouse gas emissions and the pressure on landfills from plastic waste, reduce food waste and food chemical contamination (Mohan, 2011; Álvarez-Chávez et al., 2012; Peelman et al., 2013; Nakajima et al., 2017; Guillard et al., 2018; Firoozi et al., 2021; McGauran et al., 2021). Although bioplastics are currently assumed to be safe, there is little research investigating their environmental, health and safety impacts throughout their life cycles (Álvarez-Chávez et al., 2012). One recent study highlighted that bioplastic and plant based materials may not be safer than conventional plastics (Zimmermann et al., 2020). In addition, a recent endocrine society report highlights that much more testing and development is required before we can fully address problems related to recyclability; land, biocide and water use in the production of starch containing plants for bio-based plastics; and toxic additives in plastics are solved (Flaws et al., 2020). Ideally, all new products should be safety tested prior to use, however, this is currently not the case as it is not mandatory for laboratory tests to be carried out for all packaging (European Commission, 2011a; Packaging Essential Requirements Regulations, 2015). Specific measures exist for some types of materials and substances, however, not all materials and articles, non-intentionally added substances or aids to polymerisation are required to be tested (European Commission, 2011a). Packaging which may come into contact with food should be tested to ensure they are safe for food contact, do not transfer their constituents to foods in quantities exceeding the specific migration limits and do not cause physical or other changes to the food (European Commission, 2011a). Toxicological study could help guide bioplastic product development at early stages to assist in decision making towards safer products. The production processes, generation of by-products and the biodegradability of the bioplastic could potentially pose risks to the environment, human or animal health and safety (Álvarez-Chávez et al., 2012; Nakajima et al., 2017; Ramakrishnan et al., 2018). It is important to understand the flow and production processes, chemicals used during production and any by-products generated, in order to identify any adverse impacts in all parts of the life cycles of the bioplastics (Álvarez-Chávez et al., 2012).

Additives or non-intentionally added manufacturing by-products such as fertilizers and pesticides may leach out of bioplastics into food, drink or the environment and consequently could be ingested by humans or animals (Álvarez-Chávez et al., 2012; Seltenrich, 2015). Leaching of monomers and additives from plastics into its food contents is often accelerated if the product is exposed to common-use stresses such as ultraviolet radiation in sunlight and/or via boiling or heating. Even when using materials that initially are deemed as safe, the stresses of manufacturing can change chemical structures or create chemical reactions converting a safe material or chemical into one of concern (Yang et al., 2011). However, there is limited information about the safety of bioplastics and their leachates on human health and the environment.

Migration of leachates from packaging into food is usually assessed using substitutes of foods, known as food simulants. The hydrophilic, lipophilic or amphiphilic character of the food determines the food simulant used. For example, migration into acidic foods (pH below 4.5) is measured using acetic acid 3% (w/v) (European Commission, 2011a). The migration testing is carried out under the worst foreseeable conditions of use of the plastic, therefore, the migration into food simulants is thought to overestimate the actual migration into foods (Muncke, 2014). Overall migration testing is performed on materials intended to come into contact with different food categories or a combination of food categories. Testing is performed under European standards using standardised testing conditions, in order to assess the transfer and migration of compounds into food, drink and/or the environment (European Commission, 2011a; Muncke, 2011).

The aim of this study was to investigate the toxicological safety of a range of bio-based polymers originating from a variety of renewable resources (Table 1) and in development for food and beverage packaging. In addition to the bio-based polymers, a traditional non-bio-based synthetic petroleum based polymer called polypropylene (PP) and some inorganic bio-based additives mixed with PP were also assessed. Assessment was achieved by applying migration test samples, prepared according to European test standards, to mammalian reporter gene assays (RGAs) incorporating natural sex steroid receptors capable of measuring (ant)agonism of estrogen, androgen and progestogen nuclear receptor transcriptional activity (Willemsen et al., 2004; Connolly et al., 2011; Frizzell et al., 2013). Parallel to the RGAs, the MTT assay was used to measure cellular metabolic activity as an indicator of cell viability and to monitor cytotoxic effects of test samples.

**TABLE 1:** The bio-based polymers based on their different renewable resources.

Renewable resources	Bio-based polymers
Inorganic waste products from the poultry industry	• Poultry litter ash
	• Poultry feather based polymer
	• Eggshell
Polymers directly extracted from biomass	• Thermoplastic starch material (TPS)
Polymers synthesized from bio-derived monomers	• Polybutylene succinate (PBS)
	• Polycaprolactone (PCL)
	• Polylactic acid (PLA)
	• Polybutylene adipate terephthalate (PBAT)
	• Biodolomer—PLA/PBAT blend
	• Ecovio—PLA/PBAT blend
Polymers produced directly by micro-organisms	• Polyhydroxybutyrate (PHB)

## Materials and methods

### Chemicals and reagents

Cell culture reagents Dulbecco’s Modified Eagle Medium (DMEM), general foetal bovine serum (FBS), TrypLE™ Express trypsin, trypan blue and Countess™ cell counting chamber slides were supplied by Life Technologies (Paisley, United Kingdom). The standards 17*β*-estradiol (E2) (≥97% purity), testosterone (≥98% purity) and progesterone (≥99% purity) were supplied by Sigma-Aldrich (Poole, Dorset, United Kingdom). Methanol (MeOH), dimethyl sulfoxide (DMSO), phosphate-buffered saline, charcoal-stripped FBS (10%) and thiazolyl blue tetrazolium bromide (MTT) were supplied by Sigma- Aldrich. Lysis reagents and luciferase assay system was supplied by Promega (Southampton, United Kingdom). Ultrapure water was supplied from an in-house18 MΩ Millipore water system, Millipore Ltd. (Hertfordshire, United Kingdom). The bio-based polymer samples were supplied by Dr Eoin Cunningham, Queen’s University Belfast Engineering Department, Rodenburg Biopolymers and CobelPlast, Belgium.

### Overall migration simulation

Overall migration testing was performed under European standards using standardised testing conditions (European Commission, 2011a), i.e., 24 h and 10 days at 20 and 40°C in distilled water (dH_2_O) or water of equivalent quality as shown in Table 2. MeOH was used in addition to dH_2_O as it did not dissolve any of the polymers and is normally used in the *in vitro* bioassays to dissolve the standards for application to the cells. In addition, MeOH and dH_2_O in the overall migration testing allowed for both more polar and less polar solvents to be used.

**TABLE 2:** Overall migration testing performed under standardised testing conditions (European Commission, 2011a).

Sample group	Testing time	Contact temperature (°C)	Food simulant	Intended food contact conditions
1	24 h (Contact time in worst foreseeable use of material, 6 h < t ≤ 24 h)	20°C (Contact temperature of material 5°C < T ≤ 20°C)	Distilled water or methanol	Short term storage at refrigerated conditions
2	24 h (Contact time in worst foreseeable use of material, 6 h < t ≤ 24 h)	40°C (Contact temperature of material 20°C < T ≤ 40°C)	Distilled water or methanol	Short term storage at room temperature
3	10 days (Contact time in worst foreseeable use of material, 3 days < t ≤ 30 days)	20°C (Contact temperature of material 5°C < T ≤ 20°C)	Distilled water or methanol	Any food contact at frozen and refrigerated conditions
4	10 days (Contact time in worst foreseeable use of material, 3 days < t ≤ 30 days)	40°C (Contact temperature of material 20°C < T ≤ 40°C)	Distilled water or methanol	Long term storage at room temperature or below, heating up to 70°C or up to 100°C

The bio-based polymers used in this study (Table 3) were produced using bio-based resources from waste streams, microorganisms, bio-derived monomers or biomass. The bio-based polymer pellet samples were individually weighed and added to 1 ml of ultrapure MilliQ water 18MΩ (dH_2_O) or methanol (MeOH) and conditioned in a water bath for 24 h or 10 days at 20 or 40°C in duplicate. To check the potential impact of the solvents, dH_2_O and MeOH, a blank sample was conducted in a glass vial containing only the solvent. After 24 h or 10 days of migration, the solvents dH_2_O and MeOH were evaporated under a nitrogen stream at 37°C and reconstituted in 500 µl MeOH and frozen at −20°C until use in the *in vitro* bioassays.

**TABLE 3:** Nature and supplier details of the polymer samples assessed in this study including; bio-based, inorganic bio-based mixed with a non-bio-based traditional synthetic petroleum based polymer called polypropylene, and polypropylene alone. All of the polymers were supplied pure without additional additives. Samples obtained from chickens were produced under conventional farming practices.

Bio-based polymers	Supplier
Poultry feather based polymer (organic waste)	Moplen PP Homopolymer, Ultrapolymers
Thermoplastic starch material (TPS)	AGRANA STAERKE GmbH, Conrathstraße 7, 3950 Gmuend, Austria
Polybutylene succinate (PBS)	PTT MCC BIOCHEM CO., LTD
Polycaprolactone (PCL)	BASF
Polylactic acid (PLA)	TotalEnergies Corbion bv, Stadhuisplein 70, 4203 NS Gorinchem, Netherlands
Polybutylene adipate terephthalate (PBAT)	BASF
Polylactic acid/Polybutylene adipate terephthalate blend (Biodolomer—PLA/PBAT)	Gaia BioMaterials Bunkagardsgatan 13, Helsingborg, Sverige
Polylactic acid/Polybutylene adipate terephthalate blend (Ecovio—PLA/PBAT)	BASF
Polyhydroxybutyrate (PHB)	Ecomann Biotechnology Co. Ltd. Guangdong, China
Inorganic waste additive mixed with a traditional synthetic petroleum based polymer	
Poultry litter ash/polypropylene (10:90)	Moplen PP Homopolymer, Ultrapolymers
Eggshell/polypropylene (40:60)	Moplen PP Homopolymer, Ultrapolymers
Eggshell/polypropylene (10:90)	Moplen PP Homopolymer, Ultrapolymers
Eggshell/polypropylene (0.1:99.9)	Moplen PP Homopolymer, Ultrapolymers
Traditional non-bio-based synthetic petroleum based polymer	
Polypropylene (PP)	Moplen PP Homopolymer, Ultrapolymers

### Cell culture

Three reporter gene cell lines, MMV-Luc (estrogen responsive), TARM-Luc (androgen and progestogen responsive) and TM-Luc (progestogen responsive) were previously developed by transforming human mammary gland cell lines with the luciferase gene under the control of a steroid hormone inducible promoter (Willemsen et al., 2004). The cells were routinely cultured in 75 cm^2^ tissue culture flasks (Nunc, Roskilde, Denmark) at 37°C with 5% CO_2_ and 95% humidity. The cell lines were cultured in cell culture medium containing DMEM, 10% FBS (50/500 ml medium and 1% L-glutamine). However, for culturing the MMV-Luc cell line, DMEM without phenol red was used due to the weak estrogenicity of phenol red. The cells were passaged at 80% confluency and the medium changed 2–3 times a week. Cells were detached from the flasks using TrypLE™ Express trypsin. Cell counting and viability checks prior to seeding plates were obtained using trypan blue staining and a Countess^®^ automated cell counter (Life Technologies, Paisley, Scotland). For seeding and exposures, cells were cultured in assay media containing DMEM (or DMEM without phenol red for the MMV-Luc cell line) supplemented with 10% hormone depleted serum (charcoal-stripped FBS) and 1% L-glutamine.

### Reporter gene assays

(Ant)agonist nuclear receptor transcriptional activity was assessed as previously described by Frizzell et al. (2011). Briefly, cells were seeded at a concentration of 4 × 10^5^ cells/ml into white walled, clear and flat bottomed 96-well plates (Greiner Bio-One, Frickenhausen, Germany). After 24 h, bio-based polymer samples and the relevant steroid hormone standards dissolved in methanol (MeOH) were added to the cells at a final MeOH concentration of 0.5% v:v in media. For the agonist test, the standard curves used for each cell line were: 0.00014–2.7 ng/ml E2 (MMV-Luc), 0.03–144.21 ng/ml testosterone (TARM-Luc) and 0.3–3144.6 ng/ml progesterone (TM-Luc). The positive controls used in the respective cell lines were: 0.14 ng/ml E2 (MMV-Luc), 14.5 ng/ml testosterone (TARM-Luc) and 157 ng/ml progesterone (TM-Luc). Antagonist tests were carried out by incubating the test compounds with the relative positive control for the cell line being tested. A solvent control (0.5% v:v MeOH in media) was also included for each cell line. The cells were incubated for 48 h, after which the supernatant was discarded and the cells washed once with phosphate-buffered saline. The cells were lysed with 25 μl cell lysis buffer and 100 μl luciferase substrate injected into each well and the luciferase activity measured using a Mithras Multimode Reader (Berthold, Germany). The response of the cell lines to the various compounds was measured and compared with the respective solvent and positive controls. The RGAs were performed in triplicate for each experimental point and repeated in three independent exposures.

### Thiazolyl blue tetrazolium bromide (MTT) assay

The MTT assay was performed in parallel to the RGA to measure cellular metabolic activity and to monitor the cytotoxic effects of the test compounds in the RGA cell lines. In brief, clear, flat-bottomed 96-well plates (Nunc, Roskilde, Denmark) were seeded with 4 × 10^5^ cells/ml of MMV-Luc, TARM-Luc and TM-Luc cell lines and incubated for 24 h. The test compounds at a final MeOH concentration of 0.5% and solvent control (0.5% v:v MeOH in media) were added to the cells and incubated for 48 h. The supernatant was discarded and cells washed once with 200 μl phosphate-buffered saline. Then, 50 μl of MTT solution (2 mg/ml stock in phosphate-buffered saline, diluted 1:6 in media) was added to each well and incubated for 3 h at 37°C. In this assay, viable cells convert the soluble yellow MTT into insoluble purple formazan by the action of mitochondrial succinate dehydrogenase. The supernatant was removed and 200 μl of DMSO was added to each well to dissolve the formazan crystals. The plate was incubated at 37°C for 10 min with agitation. Optical density (OD) was measured using a Sunrise spectrophotometer (TECAN, Switzerland) at 570 nm with a reference filter at 630 nm. All samples were assessed in triplicate wells and in three independent exposures. Viability was calculated as the percentage absorbance of the sample when compared with the absorbance of the solvent control.

### Statistical analysis

For the RGAs, dose-response curves were fitted with GraphPad PRISM software, version 6.0 (San Diego, CA) using the sigmoidal dose-response curve equation, Y = Bottom + (Top–Bottom)/(1 + 10 (^(LogEC50–X)^) ∗ HS), where X is the logarithm of concentration, Y the response, Bottom and Top are fixed to 0% and 100%, respectively, of the maximum response of the standard used in each test, EC_50_ concentration yielding 50% increase in maximal response and HS the hill slope. Percentage (%) response was measured by calculating the response when compared with the positive controls. All experimental points in the RGAs and MTT assays were performed in triplicate wells and repeated in three independent exposures (*n* = 3). The coefficient of variation (CV) was calculated for the three exposures; all experimental points were below 15%. Data was analysed using Microsoft Excel and GraphPad PRISM software, version 6.0. All values shown are expressed as mean ± standard error of the mean (SEM) of three independent exposures for the compounds tested. A one way analysis of variance (ANOVA) followed by Dunnett’s multiple comparison test were used to determine significant differences between samples and the corresponding controls. The mean concentrations were tested for significant difference at the 95% confidence level. Significant effects are represented by *p* ≤ 0.05 (*), *p* ≤ 0.01 (**) and *p* ≤ 0.001 (***).

## Results

The potential hormonal activity of the migration test samples were investigated at the level of nuclear receptor transcriptional activity using a panel of RGAs. Parallel to the RGAs, cellular metabolic activity was also measured using the MTT assay to monitor the cytotoxic effects of the samples. All results are summarised in Table 4.

**TABLE 4:** The bio-based polymers which leached hormonal activity as measured by RGA and the MMV-Luc (estrogen responsive) and TARM-Luc (androgen responsive) cell lines.

Bio-based polymer	Standardised testing conditions for 10 days^a^	Viability (MTT % ± SEM) ^b^ in the TARM-Luc cell line	RGA % ± SEM in the MMV-Luc cell line		Viability (MTT % ± SEM) ^b^ in the MMV-Luc cell line	RGA % ± SEM in the TARM-Luc cell line	
			Agonist^c^	Antagonist^d^		Agonist^e^	Antagonist^f^
Poultry litter ash/polypropylene (10:90)	MeOH 20°C	105.17 **±** 2.64	**3.75 ± 0.21** ***	95.36 **±** 2.57	99.09 **±** 2.84	−0.41 **±** 0.58	92.44 **±** 2.59
	MeOH 20°C	99.85 **±** 2.05	**3.82 ± 0.91** ***	91.48 **±** 1.89	100.89 **±** 1.37	0.63 **±** 0.56	99.03 **±** 3.24
	MeOH 40°C	101.43 **±** 2.36	**3.89 ± 0.40** ***	91.25 **±** 3.42	103.12 **±** 2.36	1.64 **±** 0.93	98.58 **±** 3.01
	MeOH 40°C	95.80 **±** 2.31	**4.09 ± 0.27** ***	89.82 **±** 1.98	104.86 **±** 2.48	0.75 **±** 0.67	91.99 **±** 2.96
	dH_2_0 20°C	89.39 **±** 2.86	1.49 **±** 0.98	89.97 **±** 2.87	99.41 **±** 2.23	2.13 **±** 1.00	99.25 **±** 3.25
	dH_2_0 20°C	97.71 **±** 2.94	−1.56 **±** 0.55	92.67 **±** 1.83	101.58 **±** 1.98	−1.27 **±** 0.63	97.21 **±** 2.96
	dH_2_0 40°C	94.88 **±** 2.54	−0.87 **±** 0.67	91.53 **±** 2.35	103.21 **±** 2.33	−0.34 **±** 0.53	**88.98 ± 1.63** **
	dH_2_0 40°C	93.51 **±** 2.98	0.0002 **±** 0.78	97.08 **±** 2.49	101.07 **±** 2.28	0.70 **±** 0.78	**80.51 ± 2.25** ***
Poultry feather based polymer	MeOH 20°C	102.70 **±** 1.63	1.87 **±** 0.70	95.75 **±** 2.28	103.72 **±** 2.66	0.65 **±** 0.53	95.68 **±** 3.80
	MeOH 20°C	103.39 **±** 2.03	0.57 **±** 0.64	97.89 **±** 2.79	108.06 **±** 1.62	2.97 **±** 0.81	93.40 **±** 4.10
	MeOH 40°C	101.10 **±** 2.15	2.53 ± 0.82*	90.72 **±** 2.86	92.50 **±** 1.78	1.93 **±** 0.82	91.55 **±** 2.13
	MeOH 40°C	100.43 **±** 2.00	**3.71 ± 0.75** ***	93.81 **±** 2.55	98.54 **±** 1.60	1.74 **±** 1.06	90.46 **±** 3.48
	dH_2_0 20°C	94.04 **±** 2.07	1.48 **±** 0.79	92.75 **±** 1.86	97.26 **±** 1.83	0.76 **±** 0.91	97.48 **±** 3.21
	dH_2_0 20°C	93.44 **±** 2.12	1.45 **±** 0.89	90.34 **±** 2.30	100.19 **±** 1.95	1.42 **±** 1.07	101.67 **±** 4.52
	dH_2_0 40°C	100.90 **±** 2.76	**3.49 ± 0.33** ***	91.78 **±** 3.23	102.87 **±** 3.11	0.31 **±** 0.57	100.21 **±** 4.44
	dH_2_0 40°C	110.46 **±** 2.68	**4.46 ± 0.46** ***	111.23 **±** 4.69	105.58 **±** 3.28	2.77 **±** 0.60	104.25 **±** 2.85
Eggshell/polypropylene (40:60)	MeOH 20°C	95.71 **±** 2.81	−1.09 **±** 0.88	95. 28 **±** 3.37	102.84 **±** 2.34	0.75 **±** 1.00	95.93 **±** 2.37
	MeOH 20°C	94.98 **±** 3.08	−0.62 **±** 0.89	93.52 **±** 3.58	99.39 **±** 3.33	0.94 **±** 0.91	103.01 **±** 2.54
	MeOH 40°C	99.25 **±** 2.30	−1.83 **±** 0.93	100.13 **±** 1.55	101.89 **±** 1.95	1.79 **±** 1.55	91.26 **±** 2.71
	MeOH 40°C	99.17 **±** 1.44	−0.23 **±** 0.99	96.13 **±** 4.51	102.91 **±** 2.77	1.73 **±** 1.40	91.33 **±** 2.69
	dH_2_0 20°C	91.51 **±** 3.03	−0.81 **±** 1.62	97.78 **±** 5.18	99.60 **±** 1.93	−0.41 **±** 0.37	96.91 **±** 3.56
	dH_2_0 20°C	96.04 **±** 2.50	−1.62 **±** 0.93	105.68 **±** 3.50	97.34 **±** 1.31	1.59 **±** 0.53	97.17 **±** 3.26
	dH_2_0 40°C	95.78 **±** 1.98	3.60 ± 0.28*	108.94 **±** 2.30	92.82 **±** 2.22	0.72 **±** 0.43	92.05 **±** 3.20
	dH_2_0 40°C	96.70 **±** 2.03	**4.09 ± 029** **	106.01 **±** 4.16	95.43 **±** 2.16	1.17 **±** 0.98	95.14 **±** 3.41
Eggshell/polypropylene (0.1:99.9)	MeOH 20°C	100.61 **±** 2.54	0.35 **±** 1.22	95.45 **±** 3.62	97.17 **±** 1.42	0.81 **±** 0.55	96.26 **±** 2.81
	MeOH 20°C	98.25 **±** 3.11	−0.75 **±** 0.73	99.06 **±** 2.46	104.47 **±** 2.30	−1.21 **±** 0.75	91.17 **±** 2.32
	MeOH 40°C	86.65 ± 3.08*	−0.73 **±** 1.01	100.32 **±** 2.82	98.73 **±** 1.93	−0.75 **±** 0.93	92.14 **±** 3.56
	MeOH 40°C	87.17 ± 0.95*	−1.01 **±** 0.48	102.12 **±** 2.42	105.24 **±** 2.07	0.34 **±** 0.68	90.65 **±** 3.50
	dH_2_0 20°C	90.24 **±** 1.64	−1.10 **±** 0.80	94.61 **±** 3.37	105.36 **±** 2.23	−1.92 **±** 0.77	95.81 **±** 3.21
	dH_2_0 20°C	93.90 **±** 2.56	−0.08 **±** 0.88	103.98 **±** 2.88	103.29 **±** 1.28	−0.18 **±** 0.69	100.76 **±** 3.27
	dH_2_0 40°C	94.49 **±** 2.67	−0.91 **±** 0.69	101.50 **±** 2.26	98.78 **±** 2.82	−1.98 **±** 0.75	98.79 **±** 3.20
	dH_2_0 40°C	93.30 **±** 2.70	1.55 **±** 0.66	99.12 **±** 3.42	97.94 **±** 2.03	−0.37 **±** 0.73	99.09 **±** 2.80
Polylactic acid (PLA)	MeOH 20°C	103.70 **±** 1.75	0.66 **±** 1.49	94.33 **±** 1.87	92.87 **±** 2.43	−0.12 **±** 0.79	105.04 **±** 3.51
	MeOH 20°C	100.59 **±** 1.74	1.63 **±** 1.04	93.80 **±** 1.75	90.90 **±** 1.43	0.79 **±** 0.62	98.00 **±** 2.46
	MeOH 40°C	101.61 **±** 1.46	−0.27 **±** 1.07	92.17 **±** 2.51	94.15 **±** 1.86	3.18 **±** 1.08	106.80 **±** 3.83
	MeOH 40°C	100.52 **±** 3.20	−1.19 **±** 1.07	96.80 **±** 2.98	97.80 **±** 2.51	−1.25 **±** 0.74	96.27 **±** 3.03
	dH_2_0 20°C	100.89 **±** 2.67	1.35 **±** 1.67	101.72 **±** 1.41	100.36 **±** 2.56	0.31 **±** 1.10	108.77 **±** 2.56
	dH_2_0 20°C	100.57 **±** 3.09	2.60 **±** 1.21	101.73 **±** 4.05	106.66 **±** 1.79	0.64 **±** 0.32	100.95 **±** 3.39
	dH_2_0 40°C	106.02 **±** 2.50	4.27 ± 0.57*	99.02 **±** 2.91	106.41 **±** 1.70	0.71 **±** 1.06	94.46 **±** 3.11
	dH_2_0 40°C	102.54 **±** 1.76	**5.63 ± 0.67** **	96.75 **±** 1.91	108.68 **±** 2.46	−0.16 **±** 0.88	94.03 **±** 4.11
Polypropylene (PP) (non−bio−based)	MeOH 20°C	105.50 **±** 3.13	0.07 **±** 0.92	101.69 **±** 2.54	101.47 **±** 1.33	1.86 **±** 0.69	99.06 **±** 2.48
	MeOH 20°C	103.66 **±** 3.62	3.38 **±** 0.68	101.91 **±** 3.02	102.09 **±** 1.77	−1.96 **±** 0.73	104.48 **±** 3.46
	MeOH 40°C	86.29 ± 2.38*	−1.00 **±** 0.92	94.93 **±** 2.76	98.92 **±** 1.81	−0.11 **±** 0.34	98.20 **±** 4.01
	MeOH 40°C	83.40 ± 2.79**	0.12 **±** 1.37	97.91 **±** 2.51	97.84 **±** 1.02	0.57 **±** 0.30	96.70 **±** 1.36
	dH_2_0 20°C	92.80 **±** 2.75	−0.33 **±** 1.04	96.32 **±** 3.22	96.08 **±** 1.30	0.75 **±** 0.51	99.33 **±** 3.45
	dH_2_0 20°C	94.09 **±** 2.24	0.20 **±** 0.92	98.55 **±** 3.88	99.59 **±** 1.44	0.87 **±** 0.50	100.84 **±** 2.61
	dH_2_0 40°C	86.29 ± 2.38*	−0.67 **±** 0.97	98.03 **±** 5.14	97.80 **±** 1.19	−0.83 **±** 0.36	99.53 **±** 2.44
	dH_2_0 40°C	81.87 ± 3.03**	−0.25 **±** 1.49	103.64 **±** 3.40	98.99 **±** 1.04	0.77 **±** 0.62	110.95 **±** 5.25

Viability was monitored *via* reduced cellular metabolic activity as measured by the MTT assay. The TM-Luc (progestagen responsive) cell line is not listed in the table as no activity was detected in the TM-Luc (progestagen responsive) RGA or the MTT assay. Significant results are presented in **bold** (*p < 0.05; **p < 0.01; ***p < 0.001).

a Overall migration testing performed under standardised testing conditions.

b Viability percentage is normalised against solvent control MeOH, which was set to 100%.

c Agonist response is normalised against E2 standard curve (0.0005–10 nM) and compared to solvent control MeOH, set to 0%.

d Antagonist response is compared again E2 positive control (0.5 nM) set to 100%.

e Agonist response is normalised against testosterone standard curve (0.1–500 nM) and compared to solvent control MeOH, set to 0%.

f Antagonist response is compared again testosterone positive control (50 nM) set to 100%.

*p < 0.05, **p < 0.01, ***p < 0.001.

### Metabolic activity as measured by the MTT assay

**FIGURE 1:**
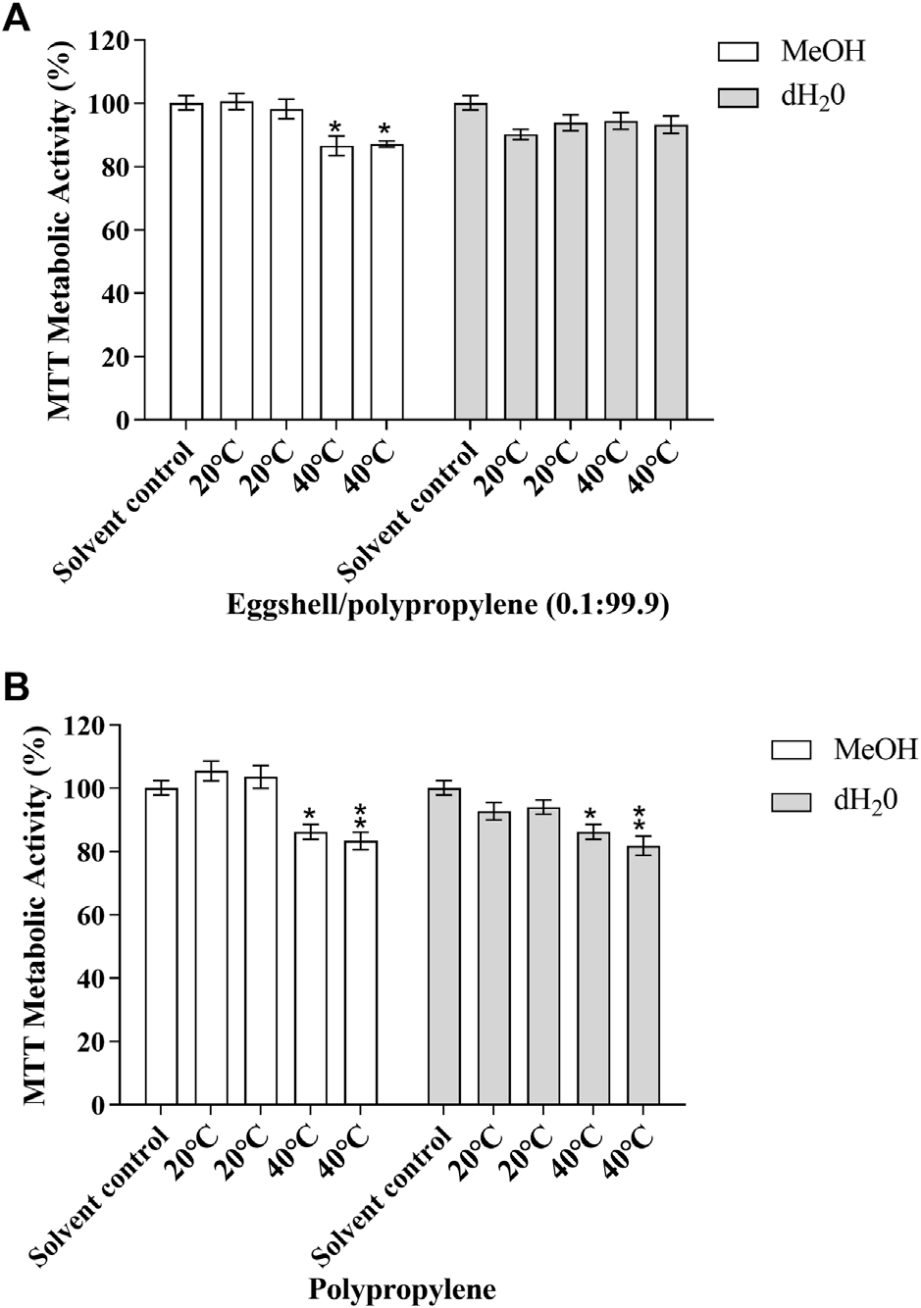
MTT metabolic activity of the MMV-Luc cell line after 48 h exposure to **(A)** eggshell/polypropylene (0.1:99.9) and **(B)** polypropylene and solvent control (MeOH 0.5%). Data is expressed as percentage of solvent control (MeOH 0.5% v:v); mean ± SEM, *n* = 3. *p* ≤ 0.05 (*) and *p* ≤ 0.01 (**).

The effects of the migration test samples on cellular viability was investigated in the MMV-Luc, TM-Luc and TARM-Luc cell lines by quantifying metabolic activity using MTT conversion. No significant effects on metabolic activity in the MMV-Luc, TM-Luc and TARM-Luc cell lines were induced by the test samples which underwent 24 h of migration testing compared to the solvent control. No significant effects on metabolic activity of the TM-Luc and TARM-Luc cell lines were induced by the test samples which underwent 10 days of migration testing compared to the solvent control (data not shown). However, metabolic activity significantly decreased in the MMV-Luc cell line (Figure 1) following treatment with eggshell/polypropylene (0.1:99.9) subjected to 10 days of migration testing at 40°C in MeOH (13.35 and 12.83%; *p* ≤ 0.05) and polypropylene (PP) after 10 days of migration testing at 40°C in MeOH (13.71 and 16.6%; *p* ≤ 0.05 and 0.01, respectively) and dH_2_O (13.71 and 18.13%; *p* ≤ 0.05 and 0.01, respectively) when compared with the solvent control (MeOH 0.5% v:v).

### Reporter gene assay (Receptor agonism)

No agonistic effects were observed at any of the standardised testing conditions of the test samples in the MMV-Luc, TARM-Luc and TM-Luc cell lines (data not shown), with the exception of four test samples in the MMV-Luc (estrogen responsive) cell line, following 10 days of migration testing (Table 5).

**TABLE 5:** The E2 equivalent estrogenic quantity (EEQ) calculated using the MMV-Luc (estrogen responsive) cell line as a measure of the estrogenic activity which leached from the bio-based polymers poultry litter ash/polypropylene (10:90), poultry feather based polymer, eggshell/polypropylene (40:60) and polylactic acid (PLA) and estimated daily human consumption of E2 EEQ *via* food packaging exposure.

Bio-Based polymer	Standardised testing conditions for 10 days	EEQ of samples (ng/ml)	Standard error of the mean (SEM)	Daily human consumption of E2 EEQs from the leachates (ng/kg body weight/day)
Poultry litter ash/Polypropylene (10:90)	MeOH 20°C	0.36	0.16	2.57
	MeOH 20°C	0.36	0.32	2.57
	MeOH 40°C	0.37	0.23	2.64
	MeOH 40°C	0.38	0.16	2.71
Poultry feather based polymer	MeOH 40°C	0.26	0.70	1.86
	MeOH 40°C	0.32	0.48	2.29
	dH_2_O 40°C	0.32	0.30	2.29
	dH_2_O 40°C	0.42	0.35	3.00
Eggshell/polypropylene (40:60)	dH_2_O 40°C	0.32	0.28	2.29
	dH_2_O 40°C	0.39	0.28	2.79
Polylactic acid (PLA)	dH_2_O 40°C	0.44	0.57	3.14
	dH_2_O 40°C	0.50	0.67	3.57

**FIGURE 2:**
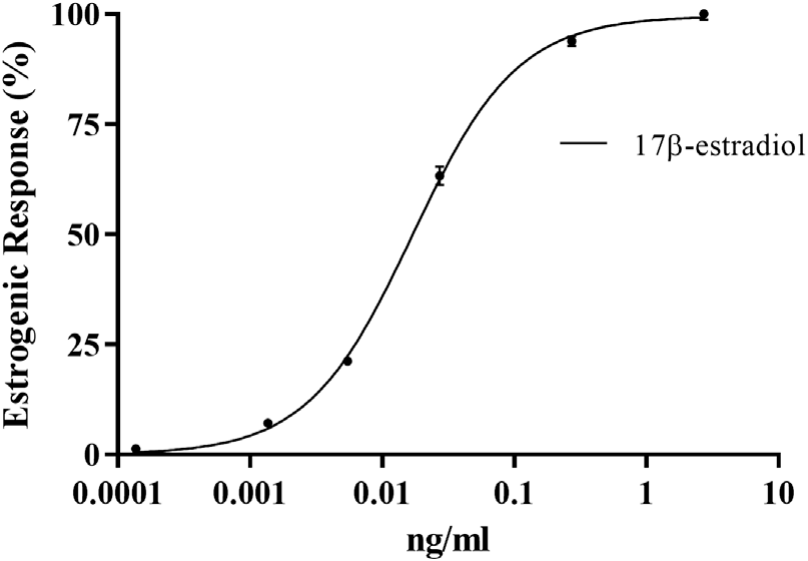
Dose-response curve of the estrogenic response of E2 with the MMV-Luc (estrogen responsive) RGA cell line. Values are means ± SEM for the three separate experiments (*n* = 3).

An estrogen dose-response curve was generated using the steroid hormone standard E2 (MMV-Luc) (Figure 2). The E2 equivalent quantity (EEQ) as a measure of the estrogenic activity of the test compounds was calculated using the sigmoidal dose-response curve equation Y = Bottom + (Top-Bottom)/(1 + 10 (^(LogEC50–X)^) ∗ HS). Where X is the logarithm of concentration, Y the response, bottom and top are fixed to 0% and 100%, respectively, of the maximum response of the standard used in each test, EC_50_ concentration yielding 50% increase in maximal response and HS the hill slope. Assuming that the average body weight of an adult is 70 kg by the EFSA, the daily human consumption levels of estrogenic compounds can be calculated based on the concentration of estrogen agonist activity (ng/kg body weight (bw)/day) = equivalent E2 concentration (ng/g) x dilution factor x daily dose (g/day)/average body weight (ABW) (kg) (EFSA, 2012; Plotan et al., 2013). The EEQ values of test samples and the calculated estrogenic activity and daily human consumption of E2 from the bio-based polymer leachates are shown in Table 5.

**FIGURE 3:**
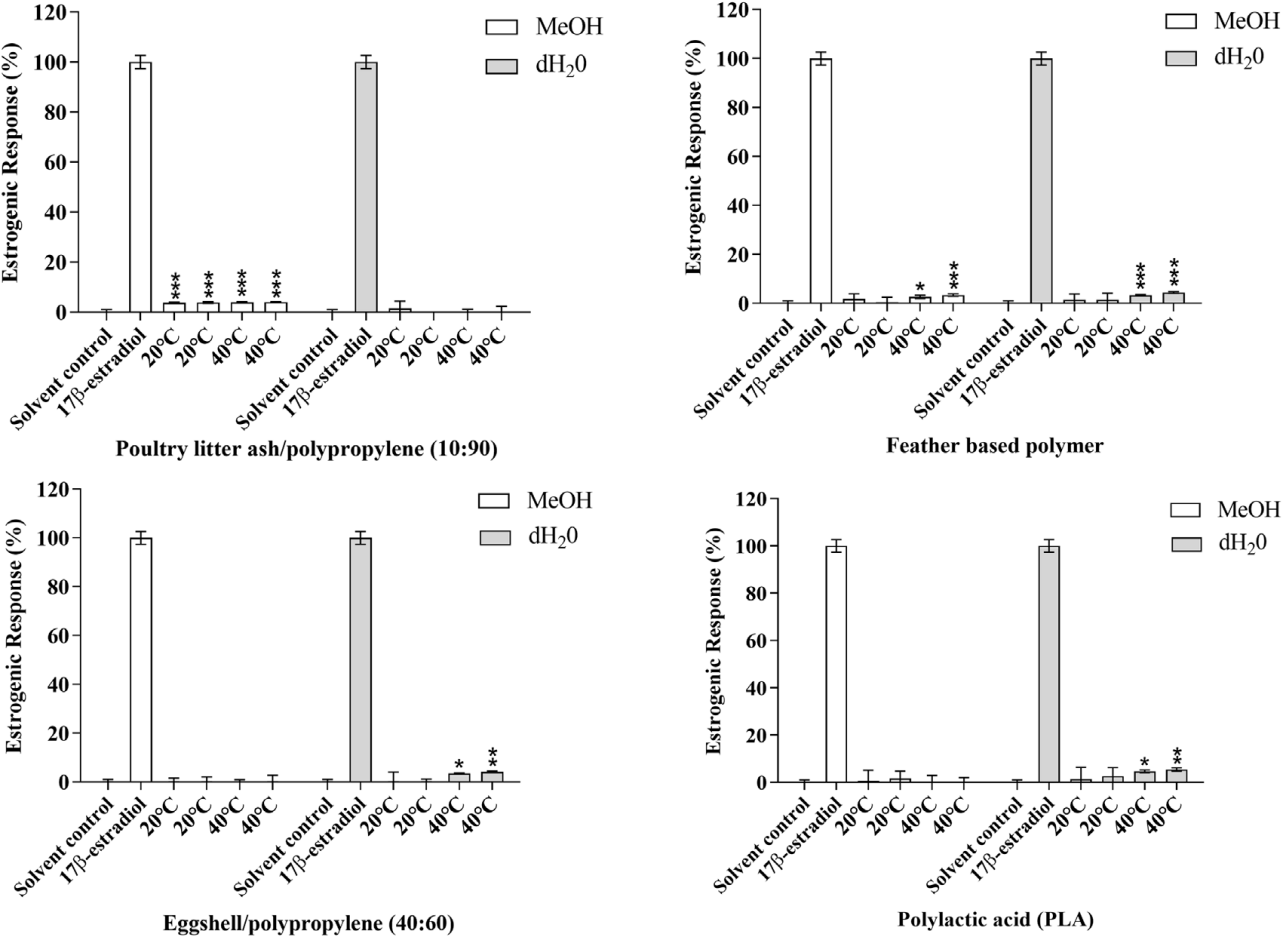
Estrogenic agonist response of **(A)** poultry litter ash/polypropylene (10:90), **(B)** poultry feather based polymer, **(C)** eggshell/polypropylene (40:60) and **(D)** polylactic acid (PLA) in the MMV-Luc (estrogen responsive) cell line. Responses measured are compared to the solvent control (MeOH 0.5%) and positive control (0.14 ng/ml E2). Response is expressed as the percentage response ±SEM, *n* = 3. *p* ≤ 0.05 (*), *p* ≤ 0.01 (**) and 0.001 (***).

After 10 days of migration testing, poultry litter ash/polypropylene (10:90) in MeOH at 20 and 40°C, poultry feather based polymer in MeOH and dH_2_O at 40°C, eggshell/polypropylene (40:60) in dH_2_O at 40°C and PLA in dH_2_O at 40°C presented responses statistically higher than the solvent control (MeOH 0.5% v:v); *p* ≤ 0.05 (*), *p* ≤ 0.01 (**) and 0.001 (***) (Figure 3). The estrogenic responses varied from 2.53%–5.63% (Table 4) with corresponding EEQ’s ranging from 0.26–0.50 ng/ml (Table 5). The bio-based polymer PLA, the most common biopolymer currently on the market (used in a variety of food contact packaging products including cups, lids, salad boxes and coatings), exhibited the strongest estrogenic activity in dH_2_O at 40°C after 10 days of migration testing; however, in comparison to E2, all of the estrogenic bio-based polymers showed relatively weak estrogenic responses in the estrogen responsive MMV-Luc cell line. All values calculated were within the acceptable daily intake (ADI) limit (0–50 ng/kg body weight) of E2 set by the JECFA (2000).

### Reporter gene assay (Receptor antagonism)

**FIGURE 4:**
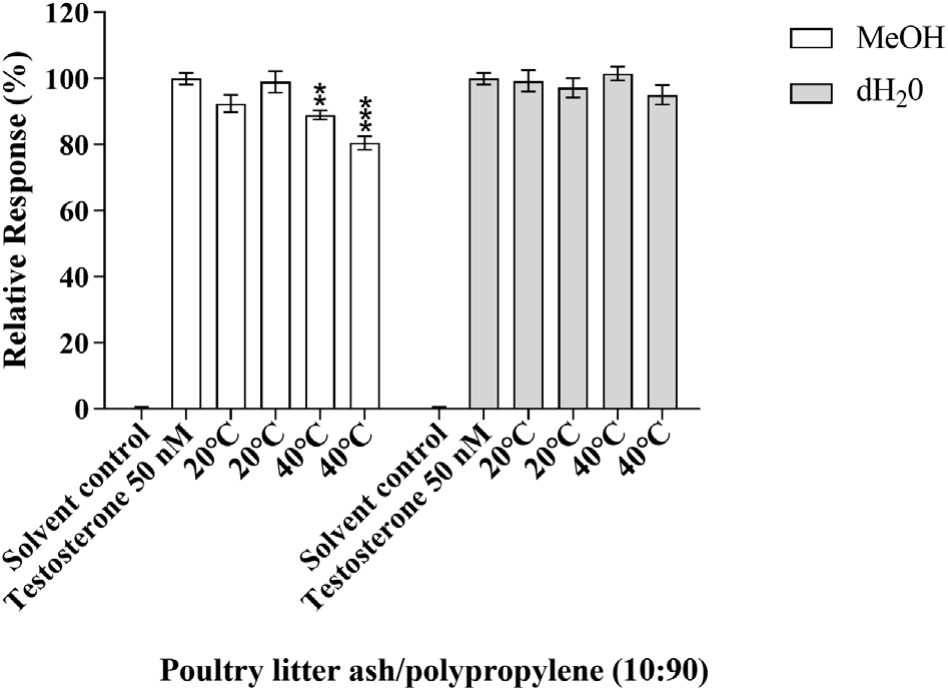
Antagonistic response of poultry litter ash/polypropylene (10:90) in the TARM-Luc (androgen responsive) cell line. Responses measured are compared to the solvent control (MeOH 0.5%) and positive control 14.5 ng/ml testosterone. Response is expressed as the percentage response ±SEM, *n* = 3. *p* ≤ 0.01 (**) and 0.001 (***).

No significant antagonistic effects were observed at any of the standardised testing conditions of the test samples in the MMV-Luc, TARM-Luc and TM-Luc cell lines (data not shown), with the exception of one test sample. After 10 days of migration testing the bio-based polymer poultry litter ash/polypropylene (10:90) in MeOH at 40°C exhibited an antagonistic effect in the TARM-Luc (androgen and progestagen responsive) cell line, with 11.02 and 19.49% reduction in androgen nuclear receptor transcriptional activity (*p* ≤ 0.01 and *p* ≤ 0.001, respectively) as shown in Figure 4. This reduction in androgen nuclear receptor transcriptional activity can be identified as true antagonism as no significant reduction in MTT metabolic activity was seen in the MTT assay (data not shown).

A summary of the results for the samples which leached compounds that exhibited a reduction in metabolic activity or an (ant)agonistic response in the MMV-Luc (estrogen responsive) or TARM-Luc (androgen responsive) cell lines are presented in Table 4. Table 4 excludes the results from the TM-Luc (progestagen responsive) cell line as no (ant)agonist activity or gross cellular cytotoxicity was detected for any of the bio-based polymers tested at any of the standardised testing conditions. The bio-based polymer leachates that did not exhibit gross cellular cytotoxicity or an (ant)agonistic response in any of the RGA cell lines at any of the standardised testing conditions as presented in Table 6. All test results for all samples with EEQs where relevant are summarised in Table 7.

**TABLE 6:** Bio-based polymer samples which showed no cytotoxic activity in the MTT assay or (ant)agonist hormonal activity in the MMV-Luc (estrogen responsive), TARM- Luc (androgen responsive) or TM-Luc (progestogen responsive) RGA cell lines at any of the standardised testing conditions.

Bio-based polymer
Eggshell/polypropylene (10:90)
Polyhydroxybutyrate (PHB)
Polybutylene adipate terephthalate (PBAT)
Thermoplastic starch material (TPS)
Polybutylene succinate (PBS)
Polycaprolactone (PCL)
Polylactic acid/Polybutylene adipate terephthalate blend (Biodolomer- PLA/PBAT blend)
Polylactic acid/Polybutylene adipate terephthalate blend (Ecovio - PLA/PBAT blend)

**TABLE 7:** Summary of results in the *in vitro* bioassay testing of all the migration test samples and mixes, including cytotoxic/metabolic effects, hormonal activity and calculated E2 equivalent quantities (EEQs).

Compound		*In vitro* bioassay results		
Category	Name	Disruption of cellular metabolic activity	Receptor agonism	Receptor antagonism
Bio-based polymers	Poultry feather based polymer	—	10 days—estrogenic—MeOH and dH_2_O at 40°C—translating to 0.26–0.42 ng of E2 equivalents per ml	—
	Thermoplastic starch material (TPS)	—	—	—
	Polybutylene succinate (PBS)	—	—	—
	Polycaprolactone (PCL)	—	—	—
	Polylactic acid (PLA)	—	10 days—estrogenic - dH_2_O at 40°C—translating to 0.44–0.50 ng of E2 equivalents per ml	—
	Polybutylene adipate terephthalate (PBAT)	—	—	—
	Biodolomer—PLA/PBAT blend	—	—	—
	Ecovio—PLA/PBAT blend	—	—	—
	Polyhydroxybutyrate (PHB)	—	—	—
Inorganic bio-additives mixed with a traditional petroleum based polymer	Poultry litter ash/polypropylene (10:90)	—	10 days—estrogenic—MeOH at 20 and 40°C—translating to 0.36–0.38 ng of E2 equivalents per ml	10 days—anti-androgenic—MeOH at 40°C—decreased androgen nuclear receptor transcriptional activity by 11.02 and 19.49%
	Eggshell/polypropylene (40:60)	—	10 days—estrogenic - dH_2_O at 40°C - translating to 0.32–0.49 ng of E2 equivalents per ml	—
	Eggshell/polypropylene (10:90)	—	—	—
	Eggshell/polypropylene (0.1:99.9)	10 days at 40°C in MeOH, corresponding to a reduction in metabolic activity of 13.35 and 12.83% in the estrogen responsive cell line	—	—
Petroleum based polymer (synthetic)	Polypropylene (PP)	10 days at 40°C in MeOH and dH2O, corresponding to a reduction in metabolic activity of 13.71 and 16.6% (for MeOH) and 13.71 and 18.13% (for dH_2_0) in the estrogen responsive cell line	—	—

—, no detected activity.

## Discussion

This study investigated the potential endocrine disrupting effects of bio-based polymers at the level of estrogen, androgen and progestagen nuclear receptor transcriptional activity using RGAs. Parallel to RGAs, metabolic activity was measured using the MTT assay, as an indicator control of sample gross cellular cytotoxicity. All cytotoxic and hormonal leachate activity observed in the study is summarised in Table 7.

The study revealed that eggshell/polypropylene (10:90), PHB, PBAT, TPS, PBS, PCL and PLA/PBAT blends did not induce any hormonal or cytotoxic activity at any of the standardised testing conditions. Therefore, they are potentially safer candidates for further development in the bioplastic industry, particularly for food packaging applications.

After 10 days of migration testing at 40°C, eggshell/polypropylene (0.1:99.9) in MeOH and the non-bio-based PP in MeOH and dH_2_O decreased metabolic activity but only in the estrogen responsive MMV-Luc cell line. This cell line specific effect may be due to the robustness of the differing RGA parent cell lines, the MCF-7 cell line is the parental lineage for the MMV-Luc cell line while T47D is the parental lineage for the TM-Luc and TARM-Luc cell lines. The traditional synthetic polymer PP is commonly used in the manufacture of food containers and has previously been described as a safe non-EDC-related material (Bang et al., 2012; Chung et al., 2013) with cytotoxic effects (Hwang et al., 2019; Zimmermann et al., 2019). Using a CCK-8 colorimetric assay to measure both cell viability and proliferation, Hwang et al. (2019) found that direct contact of PP particles and PP dispersed in medium had cytotoxic effects on peripheral blood mononuclear and murine macrophages Raw 264.7 cells, concluding chemicals may be released from PP particles when they are dissolved in the medium or in direct contact with cells. Zimmermann et al. (2019) found that food contact materials (FCMs) containing PP, such as yoghurt cups and gummy candy packaging, had varied baseline toxicity and cytotoxicity, with some inducing toxicity in the Microtox assay by inhibiting bioluminescence in the bacterium *Aliivbrio fischeri* and some inducing cytotoxicity in the AREc32 assay and yeast-based RGA cell lines. The toxicity of PP based FCMs is dependent on the chemical composition of the packaging, additives such as the antioxidant butylated hydroxytoluene and the plasticiser tributyl acetylcitrate have been reported to induce cytotoxicity in ToxCast data (Zimmermann et al., 2019). In the current study, a reduction in metabolic activity was only observed at test conditions of the higher temperature (40°C) and longer period (10 days). Similarly, Alin and Hakkarainen (2011) found that heating PP based FCMs lead to an increase in degradation of antioxidants, which led to increased migration of PP into the food simulants. It would be of future interest to identify the cytotoxic PP leachate(s) responsible, but this will require further work and analytical methods capable of detecting unknowns. Overall, these results indicate that PP is not suitable for FCM development and eggshell/polypropylene (0.1:99.9) may only be suitable for use with more polar solvent like based foods stored and used at low temperatures. Alternatively, these formulation profiles could be developed for non-food contact applications.

In contrast, eggshell/polypropylene (40:60) and eggshell/polypropylene (10:90) which contain lower levels of PP (60% and 90%, respectively) did not reduce metabolic activity at any of the standardised test conditions. In addition, the cytotoxic PP leachate(s) readily leached into MeOH but only into dH_2_O when eggshell was at very low concentrations of 0.1% or was not present. This suggests that eggshell may bind the toxicant at a higher affinity in dH_2_O when compared to in MeOH. This specific binding could provide a beneficial clean up tool in many packaging formulations. Indeed it would be very interesting future work to add eggshell to a polymer known to leach EDCs and assess if the introduction of calcium carbonate can bind and clean up the EDCs.

Most plastic products leach estrogenic chemicals, highlighting the potential for endocrine disrupting leachates to be present in food or drink products (Yang et al., 2011; Zimmermann et al., 2020). The current study demonstrated that after 10 days of migration testing, four of the test samples presented estrogenic activity as outlined in Table 4, including poultry litter ash/polypropylene (10:90), poultry feather based polymer, eggshell/polypropylene (40:60) and PLA. Whilst it is desirable to have zero hormonal activity, detected estrogenic activity was at low levels, ranging from 2.67% to 5.47% relative response in the ER RGA with corresponding EEQ’s ranging from 0.26 to 0.50 ng/ml. Assuming that the average body weight of an adult is 70 kg, the exposure level of estrogenic compounds via food or drink packaged in the bio-based polymers can be calculated (EFSA, 2012; Plotan et al., 2013). Plotan et al. (2014) calculated the exposure levels to estrogenic compounds based on the concentration of estrogen agonist activity (ng/kg body weight (bw)/day) = equivalent E2 concentration (ng/g) x dilution factor x daily dose (g/day)/average body weight (ABW) (kg). However, the daily doses of the products are unknown as the bio-based polymers are in the developmental stage and have not yet been used in the industry. Therefore, assuming an adult consumed an average daily intake of 500 g or 500 ml of food or drink packaged in the bio-based polymers, the exposure level of estrogenic compounds ingested can be calculated, ranging from 1.86–3.57 ng/kg bw/day (Table 5). These exposure levels are lower than the typical adult eating an omnivorous diet, which was estimated as 23 ng/person/day EEQ of endogenous estrogens. However, this is an estimated value which does not take into consideration the contribution of phytoestrogens or other estrogens which have no estrogenic potency or occurrence data available (Caldwell et al., 2010; Plotan et al., 2013). JECFA (2000) established that the ADI for E2 is up to 50 ng/kg bw/day. Therefore, it may be concluded that based on the assumed exposure level of estrogenic compounds ingested, the level of estrogenic activity of the bio-based polymer leachates do not pose a safety risk for consumers. Similarly to our findings suggesting that using inorganic waste additives such as eggshell can remove cytotoxic leachates from polymers such as PP, it may also be possible in the future to manipulate biopolymer recipes to reduce or completely eliminate this low level hormonal activity.

The estrogenic activity detected in the test samples was minimal compared to the estrogenic activity of BPA which has been identified to leach out of plastic products (Kuruto-Niwa et al., 2005; Grignard et al., 2012; Bittner et al., 2014; Hu et al., 2016). The European Commission set a specific migration limit for BPA; 600 μg/kg of food before banning the use of BPA in the manufacture of polycarbonate infant feedings bottles (European Commission, 2004; 2011b). BPA was reported to migrate out of polycarbonate plastic baby bottles at a migration level of 1.83 μg/kg (0.008 µM) (Simoneau et al., 2011). Le et al. (2008) found BPA leaches out of polycarbonate drinking bottles at room temperature at a concentration of 1 ng/ml (0.004 µM), however, after exposure to 100°C the concentration of BPA (7.67 ng/ml; 0.03 µM) leaching out of plastic bottles increased. BPA at relative leaching concentrations may be associated with hormone responsive breast cancers which are likely mediated by BPA’s estrogenic activity; activating ERα-mediated signalling and subsequently alter response in gene transcription and expression in breast epithelial cells (Weng et al., 2010). In addition, an association between BPA (1.66 ng/ml) at relative leaching concentrations and the risk of recurrent miscarriages in humans was identified by Shen et al. (2015). Previous work (Harper, 2020) has demonstrated that BPA (0.002–20 µM), bisphenol-F (BPF) (0.002–200 µM) and bisphenol-S (BPS) (0.002–20 µM) exhibited estrogenic activity at concentrations relevant to the tolerable daily intake (TDI) of 4 µg BPA/kg body weight/day for a 70 kg person (dose equates to ∼0.02 µM) (EFSA, 2015). The estrogenic responses varied from 14.91% to 54.23% for BPA, 17.1%–131.2% for BPF and 16.23%–79.22% for BPS with corresponding EEQ’s ranging from 1.354 to 7.838 ng/ml for BPA, 1.58–736.47 ng/ml for BPF and 1.49–22.83 ng/ml for BPS. The EEQ calculation allows the estrogenic potency of the compounds to be determined (Vega-Morales et al., 2013). Previous studies have revealed that BPA, BPF and BPS *in vitro* have similar estrogenic potencies which are 100,000-fold less potent than 17*β*–estradiol (Grignard et al., 2012; Rochester and Bolden, 2015; Mesnage et al., 2017). In comparison to the bisphenols, the test samples which induced a weak estrogenic response in the current study, had significantly lower estrogenic potencies through receptor activity, ranging from 3 to ∼2800 times less potent. The minimal estrogenic activity and potency of the bio-based polymer leachates investigated in the current study may suggest that the bio-based polymers could be a safer alternative to plastic packaging using compounds such as BPA.

Poultry feather based bio-polymer is composed of propanol glycol (28%), a catalyst included in the recipe (sodium sulphite, 1–2%) and the protein keratin which contains high amounts of the amino acid cysteine with thiol groups (Shalini and Singh, 2009; Xu et al., 2017). The MMV-Luc cell line is specific for the detection of ER transcriptional activation and endogenously expresses both Erα and Erβ, but predominantly Erα (Willemsen et al., 2004; Eze et al., 2019). The ER is a DNA-binding protein that functions as a transcription factor in response to estrogen activation of the protein. The DNA-binding domain of the receptor contains two zinc fingers; each finger has a zinc atom coordinated to 4 cysteines (Garcia-Morales et al., 1994). Therefore, the cysteine and thiol groups in the keratin protein may disrupt the hormone-binding domain of Erα (Zhou et al., 2013). Estrogenic activity of the poultry feather based bio-polymer was only observed at the higher temperature of 40°C. Aranberri et al. (2017) found that chicken feathers used for biodegradable polymeric materials had a large low-temperature endothermic peak at 77°C; this peak ranged from 30–130°C and identified the amount of bound water in the keratin structure which can be referred as the “denaturation” temperature. Therefore, keratin protein may have started to unfold and loosen, subsequently leading to the migration of estrogen-like compounds or additives out of the bio-based polymer (Groot and Bakker, 2016; Lapidus, 2017).

PLA exhibited the strongest estrogenic activity out of all the bio-based polymers tested with an EEQ value of 0.50 ng/ml. Zimmermann et al. (2019) found that the FCMs with PLA, yoghurt cup and vegetable tray, activated the human ERα using a yeast-based estrogen reporter gene assay. In agreement with our findings, Zimmermann et al. (2019) observed low estrogenic activity for PLA, 5.47% and 0.34%, respectively. ToxCast data identified numerous compounds including fatty acids; dodecanoic acid (lauric acid), octadecanoic acid (stearic acid), n-hexadecanoic acid (Palmitic acid) and oleic acid which can leach out of PLA plastics and induce estrogenic activity (Zimmermann et al., 2019). Studies have found fatty acids to modulate ERs by alterations in E2 binding to receptors and/or by cleaving native ERs (Borras and Leclercq, 1992; Prinsloo and van Aswegen, 2002). Borras and Leclercq, 1992 found that unsaturated fatty acids can disrupt ER action through an intracellular interaction between ER and unsaturated fatty acids acting as second messengers in regulation of cellular functions or through transmembrane modulation of phosphokinases and/or phospholipases implicated in ER mechanism of action. Yang et al. (2011) reported estrogenic activity (>15% of relative maximum response to E2) of plastic packaging made from PLA leached into saline using the MCF-7 cell proliferation assay. The results from Yang et al. (2011) correlate to the results found in the current study as PLA leached into more polar solvents, dH_2_O and saline, and exhibited estrogenic activity whilst no estrogenic activity was found for PLA tested in less polar solvents, MeOH and ethanol. Polar solvents have been identified to increase the degradation and release of migrant compounds in PLA packaging due to polymer hydrolysis. Lactic acid is the lone monomer in PLA and can migrate either as lactic acid or joined to dimmers and oligomers produced by PLA hydrolysis (Fortunati et al., 2012). There has been a reduction in the application of PLA in food packaging, as PLA has demonstrated low thermal stability and poor barrier properties (Fortunati et al., 2012).

Poultry litter ash/polypropylene (10:90) presented both estrogenic and anti-androgenic hormonal activity. In agreement with our findings, Bittner et al. (2014), identified that PP in the absence of any additional chemicals added during manufacturing of BPA-free polycarbonate plastics exhibited no detectable estrogenic activity in MCF-7 cells. We also found no (anti)androgenic activity for PP in this study. Consequently, the hormonal activity must originate from the poultry litter ash element. The composition of poultry litter ash/polypropylene (10:90) includes polypropylene, potassium sulphate, potassium chloride, calcium phosphate, sodium fluoride and phosphorus pentoxide. Fluoride has previously been identified as an EDC, altering normal endocrine function and response (National Research Council, 2006) and sodium fluoride has also been found to act as an EDC at low doses (Vandenberg et al., 2012), inhibiting insulin secretion plus the parathyroid and thyroid hormones. Poultry litter ash/polypropylene (10:90) also leached a substance which antagonised androgen receptor (AR) activation in the less polar solvent, MeOH, but not the more polar solvent, dH_2_O, at 40°C after 10 days of migration testing. Sodium fluoride dissolves more freely in MeOH as the temperature of the solvent increases, therefore, sodium fluoride may have leached out of the poultry litter ash/polypropylene (10:90) into MeOH at 40°C (Germuth, 1931). Sodium fluoride has also been reported to inhibit AR mRNA expression in Sertoli cells in mice, reducing AR protein and gene expression in the testis, therefore, negatively affecting male fertility (Huang et al., 2008). The decrease in AR transcriptional activity observed in the current study may be due to the effect of fluoride on 90-kDa heat-shock protein (HSP90) which may reduce transforming growth factor receptor-2 (TGFR-2) levels, and suppress TGF-β signalling activity, via upregulated cyclins D1 (CCND1) (Knudsen, et al., 1999; Le et al., 2017). Previous studies have also identified that the plastic leachate BPA can directly bind to the AR acting as an antagonist of the AR *via* inhibiting the actions of endogenous androgens and transcription of AR target genes at concentrations above and below the total daily intake (4 μg/kg body weight/day for a 70 kg person) (Lee et al., 2003; Xu et al., 2005; Sun et al., 2006; Bonefeld-Jørgensen et al., 2007; Teng et al., 2013; Ma et al., 2019). BPA can reduce AR transcriptional activity in mouse Sertoli 15p-1 cells with an IC_50_ 0.08 µM and can decrease AR transcriptional activity by more than 20% at relative leaching concentrations 0.01 µM (Lee et al., 2003). The antagonistic effects of BPA on the AR may lead to adverse effects on the male reproductive system such as decreasing sperm counts, interfering with sperm motility and reducing reproductive organ weights (Xu et al., 2005). The reduction in AR transcriptional activity following exposure to poultry litter ash/polypropylene (10:90) was less than that of relative leaching concentrations of BPA. Therefore, the bio-based polymers investigated in the current study may potentially be safer alternatives to plastic packaging using compounds such as BPA and potentially safer candidates for further development in the bioplastic industry.

Estrogenic activity was detected in the bio-based polymer with the highest ratio of eggshell to polypropylene (40:60). The composition of chicken eggshell consists of 95% calcium carbonate, eggshell membrane, phosphorous, magnesium, strontium and traces of sodium, zinc, iron, lead, manganese and copper (Mora, 2003; Rovenský et al., 2003; Ummartyotin and Tangnorawich, 2015; Mohammadzadeh et al., 2019; Ajayan et al., 2020). The leachate compound(s) responsible for inducing the estrogenic activity of the poultry eggshell is unknown. Eggshells may contain biologically active compounds such as heavy metals and amino acids rich proteins such as histidine, arginine and aspartic acid (Nakano et al., 2003; Nagamalli et al., 2017). Information regarding the estrogenic activity of the metal strontium is limited. However, previous studies have found that metals such as cadmium, mercury, arsenic, lead, manganese and zinc can affect hormone levels, hormone production, gene transcription activity of steroid receptors and mimic hormone action (Garcia-Morales et al., 1994; Lafuente et al., 2003; Smida et al., 2004; Srivastava et al., 2004; Pine et al., 2005; Bodwell et al., 2006; Agusa et al., 2007; Iavicoli et al., 2009). Some metals have been classified as metalloestrogens due to their ability to interfere with the action of estrogenic hormones (Safe, 2003). Metals and amino acids have the ability to disrupt the action of E2, ERs and DNA due to the participation of zinc-fingers motifs in processes such as ER dimerization and binding with DNA. These zinc-fingers motifs have cysteine and histidine with high affinity for metals. Studies have shown that metals and amino acids are capable of binding to the DNA-binding domain of the ER zinc finger motifs and disrupting the binding capacity of some transcription factors with DNA (Zawia et al., 1998; Reddy and Zawia, 2000; Razmiafshari et al., 2001; Torre et al., 2011). Metals including aluminium, antimony, arsenite, barium, cadmium, chro-mium (Cr(II)), cobalt, copper, lead, mercury, nickel, selenite, tin and vanadate are capable of binding to the ligand binding domain of the Erα, enhancing the agonist activity of E2 to their ERs, therefore, acting as estrogen agonists in test systems including affinity chromatography, MCF7 proliferation and reporter genes (Darbre, 2006). Consequently, the presence of metals in eggshells may have the potential to induce estrogenic activity in the MMV-Luc cell line *via* a similar action to that of metalloestrogens.

The migration testing employed in the current study was carried out under the worst foreseeable use of the plastic conditions, therefore, the migration of leachates into food simulants is thought to overestimate the actual migration into foods (European Commission, 2011a; Muncke, 2014). Consequently, it should also be noted that in the current study the cytotoxic and hormonal activity of the leachates may be overestimated.

The endocrine disrupting potential of FCMs is dependent on the chemical composition of the plastic packaging, however, manufacturers of plastic products typically do not list their chemical composition (Yang et al., 2011). Polymers and bio-polymers are produced with small quantities of various additives such as antioxidants and plasticisers which are physically but not chemically bound to the polymeric structure, therefore, these additives can leach from the plastic products at very low (e.g., nanomolar to picomolar) concentrations which individually or in combination can cause adverse health effects (Flaws et al., 2020). The manufacturing process of plastic uses many monomers and additives that may exhibit estrogenic activity or potentially decrease nuclear receptor transcriptional activity due to physicochemical properties that enables them to bind to nuclear receptors (Yang et al., 2011). Leaching of additives and monomers from plastics into its contents such as food is often accelerated if the product is exposed to common-use stresses such as ultraviolet radiation in sunlight and/or moist heat via boiling or dishwashing. However, many biodegradable polymers are sensitive to water and moisture so will not be suitable for use in dishwashers. Even when using materials that initially are not known to leach hormonally active compounds, the stresses of manufacturing can change chemical structures or create chemical reactions to convert a non- hormonally active compound into a chemical with endocrine disrupting activity (Yang et al., 2011).

Polarity and solubility properties are important factors for migration due to the interactions between the polymer, migrants and the food simulant. If the migrant has poor solubility in the food simulant it will be retained in the polymer matrix, while a simultaneous effect based on a high affinity between food simulant and the polymer may lead to absorption by the polymer matrix. In addition, absorption of solvents may cause swelling of the polymer matrix, therefore, enlarging the gaps between the molecules, leading to the migration of small particles and degradation products (Zygoura et al., 2011; Fortunati et al., 2012). Results also differed for the same bio-based polymer under the same standardised testing conditions between leaching into more polar dH_2_O and less polar MeOH. Therefore, the use of more polar and less polar solvents should be assessed to ensure the ability to detect more polar and less polar leachate activity as plastic containers may hold either type of liquid or a liquid that is a mixture of more polar and less polar solvents (e.g., milk) (Yang et al., 2011).

Solubility, temperature and exposure time are significant factors controlling the migration of leachates. In agreement with our study, Muncke (2014) also found that the extent of chemical migration from food packaging into food and food simulant is highly correlated with temperature and duration of exposure. The migration of hormonally active leachates out of the poultry feather based polymer, eggshell/polypropylene (40:60), PLA and poultry litter ash/polypropylene (10:90) occurred after migration testing was implemented under conditions representing long term storage at room temperature or below or heating up to 100°C. In contrast, under conditions representing short term storage at room temperature or below, none of the bio-based polymers appeared to leach substances with hormonal activity.

## Conclusion

Endocrine disrupting and toxic packaging leachates are a major health concern for the consumer and environment. Whilst all the bio-based polymers investigated in this study presented either negligible or less hormonal leachate activity in comparison to BPA, ideally it is desirable to develop packaging with zero toxicological hazard. It should also be noted that whilst the current study carried out initial investigations at the level of estrogen, androgen and progestogen nuclear receptor transcriptional activity, further investigations should also investigate other endocrine-related pathways, acting through different mechanisms of action.

The traditional petroleum based polymer PP did not present hormonal activity but did leach a potentially toxic compound which reduced metabolic activity within our estrogen responsive MMV-Luc cell line. The bio-based polymers eggshell/polypropylene (10:90), PHB, PBAT, TPS, PBS, PCL and PLA/PBAT blends did not present any hormonal or cellular cytotoxicity activity at any of the standardised testing conditions and thus may be potentially safer candidates for further development for the food contact packaging industry. The poultry feather based polymer and PLA and the formulations eggshell/polypropylene (40:60) and poultry litter ash/polypropylene (10:90) presented estrogenic activity, with the latter also presenting anti-androgenic activity. These developmental polymers and their formulations may be more suitably streamed towards further development in non-food packaging areas.

The major finding in this study is that altering formulations and their constituent levels may eradicate toxic and or hormonal activity. For example, the estrogenic and toxic activity observed in the eggshell and PP formulations was eliminated in the eggshell/polypropylene (10:90) formulation which consists of relatively lower eggshell and PP concentration as compared to the other formulations or PP alone. The mechanism by which this happens remains to be elucidated but effects may be diluted with compound reduction or alternatively eggshell may bind and eliminate the toxic elements of PP. Similarly the estrogenic activity observed in PLA was eliminated in PLA/PBAT blends.

In conclusion, this study is the first to demonstrate that combining toxicological analysis with the development of new bio-based and or manipulating mixtures of inorganic additives with a traditional synthetic polymer may eradicate hormonal and toxic leachates from packaging material, thus providing a useful approach for safer packaging development.

## Data Availability

The original contributions presented in the study are included in the article/supplementary material, further inquiries can be directed to the corresponding author.
